# The Peripheral Olfactory Repertoire of the Lightbrown Apple Moth, *Epiphyas postvittana*

**DOI:** 10.1371/journal.pone.0128596

**Published:** 2015-05-27

**Authors:** Jacob A. Corcoran, Melissa D. Jordan, Amali H. Thrimawithana, Ross N. Crowhurst, Richard D. Newcomb

**Affiliations:** 1 School of Biological Sciences, University of Auckland, Auckland, New Zealand; 2 The New Zealand Institute for Plant & Food Research Ltd, Auckland, New Zealand; United States Department of Agriculture, Beltsville Agricultural Research Center, UNITED STATES

## Abstract

The lightbrown apple moth, *Epiphyas postvittana* is an increasingly global pest of horticultural crops. Like other moths, *E*. *postvittana* relies on olfactory cues to locate mates and oviposition sites. To detect these cues, moths have evolved families of genes encoding elements of the peripheral olfactory reception system, including odor carriers, receptors and degrading enzymes. Here we undertake a transcriptomic approach to identify members of these families expressed in the adult antennae of *E*. *postvittana*, describing open reading frames encoding 34 odorant binding proteins, 13 chemosensory proteins, 70 odorant receptors, 19 ionotropic receptors, nine gustatory receptors, two sensory neuron membrane proteins, 27 carboxylesterases, 20 glutathione-S-transferases, 49 cytochrome p450s and 18 takeout proteins. For the odorant receptors, quantitative RT-PCR corroborated RNAseq count data on steady state transcript levels. Of the eight odorant receptors that group phylogenetically with pheromone receptors from other moths, two displayed significant male-biased expression patterns, one displayed significant female-biased expression pattern and five were expressed equally in the antennae of both sexes. In addition, we found two male-biased odorant receptors that did not group with previously described pheromone receptors. This suite of olfaction-related genes provides a substantial resource for the functional characterization of this signal transduction system and the development of odor-mediated control strategies for horticultural pests.

## Introduction

In moths, the ability to detect and respond to odors is essential for finding potential mates, food, and hosts on which to lay their eggs. For moths, odor and pheromone reception predominantly takes place within specialized hairs known as sensilla on their antennae. Each sensillum contains one to three olfactory sensory neurons that extend dendrites into the lymph within the hair. Odorants enter this lymph through wax-filled pores in the external surface of the sensillum and are transported through the lymph to receptors on the surface of the dendrites, before being broken down to reset the signaling system [1,2].

Proteins involved in these peripheral signaling events have been identified from moths, and include families of proteins involved in binding and transporting odorants, reception and signaling, and system resetting (see [3] for review). Members of all these families have been proposed as targets against which to develop novel, olfactory-mediated pest control strategies.

Odorant Binding Proteins (OBPs) form a large family of small (~15 kDa) hydrophilic carrier proteins that contain two-four disulphide bridges. Within the OBPs are subfamilies specialized in carrying general odorants (General Odorant Binding Proteins, GOBPs; Antennal Binding Protein Xs, ABPXs) and sex pheromone components (Pheromone Binding Proteins, PBPs). Other families of carrier proteins found in the antennae include the smaller Chemosensory Binding Proteins (CSPs) and the larger Takeout proteins (TOs). In the antennae, members of these families are thought to be expressed in the accessory cells and secreted into the sensillum lymph. Structural and binding studies have demonstrated that members of these families are capable of binding small molecules including many odorants and pheromones [4–8].

Three different families of integral membrane proteins have been implicated in the reception of odors. These include members of the odorant receptor (OR) and ionotropic receptor (IRs) families, which are both ligand-gated ion channels [9,10], and the sensory neuron membrane proteins (SNMPs). The ORs and IRs are represented by large families of proteins, whereas only two SNMPs are typically present in moths. ORs are seven-transmembrane-domain non-selective cation channels with intracellular N-termini [11–13]. They include a common obligate co-receptor known as Orco that is required for reception but is not tuned to odors [13]. This co-receptor pairs with tuning receptors to detect a range of different odorants. These OR-Orco complexes are present in the dendritic membrane of sensory neurons housed in sensilla trichodea and basiconica. In comparison, IRs are three-transmembrane-domain proteins that typically detect volatile acids and similar compounds and are found in sensory neurons within sensilla coeloconica [14]. They too form multimeric receptor complexes. SNMPs are members of the CD36 class of membrane proteins and are predicted to contain two-transmembrane regions [15]. The exact role of SNMPs remains unclear.

Pheromone receptors (PRs) form a specialized subset of ORs in moths. They have been identified in many species, mainly from members of the Noctuidae [16,17], Bombycidae [18,19], Saturniidae [20] and Crambidae [21,22]. To date all PRs fall into a single phylogenetic clade within the OR family and generally display higher levels of expression in RNA in the antennae of males than those of females (male-biased expression). Compared with other ORs, receptors from this clade seem to evolve faster, especially compared with Orco [23], with evidence some members are physically clustered within the genome [24].

Members of three families of enzymes have been implicated in the removal of odorant and pheromone compounds in the sensillum lymph to reset the odorant reception system. These odorant degrading enzymes (ODEs) include carboxylesterases (CXEs), glutathione-S-transferases (GSTs) and cytochrome p450s (CYPs). Members of all three families are expressed within moth antennae [25]. In particular, certain CXEs are capable of hydrolyzing acetate sex pheromone components of *Antheraea polyphemus* at an exceptionally rapid rate [26].

The methods used to isolate members of these families have improved significantly with the advent of next generation sequencing technology, both in sequencing cDNA and also directly sequencing genomes. More and more projects are using these technologies to isolate and sequence transcripts from moth antennae, while the complete genomes of an increasing number of species within the Lepidoptera are also being sequenced [27–30].

The lightbrown apple moth, *Epiphyas postvittana*, is a horticultural pest in Australia and New Zealand [31]. *E*. *postvittana* is a member of the leafroller family Tortricidae, which also contains a number of other horticultural pests including codling moth (*Cydia pomonella*) and the oriental fruit moth (*Grapholita molesta*). Originating from Australia, *E*. *postvittana* has subsequently become established in New Zealand, Hawaii, California and parts of Europe [31,32]. The moth is primarily a pest of pipfruit, but is also found in high numbers in vineyards [31]. As well as insecticides, odor-mediated strategies such as mating disruption have been employed to control its numbers. The sex pheromone for *E*. *postvittana* has been identified and is mainly comprised of (*E*)-11-tetradecenyl acetate and (*E*,*E*)-9,11-tetradecadienyl acetate [33], with further minor components also being identified more recently [34]. Electrophysiological studies have demonstrated that, as well as the major pheromone components [35], antennae of *E*. *postvittana* can detect many terpenes, esters, alcohols and aldehydes [36].

Some of the elements of the molecular machinery have already been identified and characterized from the periphery of *E*. *postvittana* antennae [37–40]. These include PBPs, GOBPS, ABPXs and CSP carrier proteins, some ORs, and ODEs from the CXE, GST and CYP families. Pheromone Binding Proteins 1 and 3 from *E*. *postvittana* (EposPBP1, 3) are more highly expressed in male antennae than in female antennae, whereas EposPBP2 shows the opposite bias in expression [37]. EposPBP1 is capable of binding the major sex pheromone component and displays a high degree of allelic diversity [40]. Also showing male-biased expression in *E*. *postvittana* antennae is the takeout protein EposTO1 [37]; however, an *in vivo* ligand has yet to be identified for this carrier [7,8]. Three ORs have been isolated from *E*. *postvittana* [38]. These include the Orco ortholog EposOR2; a receptor, EposOR3, with high affinity for monoterpenes [38,41] that is conserved across the Lepidoptera; and a third (EposOR1) that falls within the pheromone receptor clade.

Here we use next generation sequencing technologies and bioinformatic analyses to isolate further candidate elements of the peripheral olfactory repertoire from *E*. *postvittana*. We identify large families of carrier proteins, receptors and ODEs. Within the ORs we identify two groups of male-biased receptors, with one found outside the pheromone receptor clade.

## Methods

### Insect Rearing

*E*. *postvittana* were obtained from a colony maintained at the New Zealand Institute for Plant & Food Research Ltd, Auckland, New Zealand. Larvae were reared on a general all-purpose diet [42]. Moth antennae and bodies (no head or wings) were removed from two- to three-day-old, cold-anesthetized adults using forceps and immediately frozen in liquid nitrogen and stored at -80°C.

### Next Generation Sequencing

Total RNA was extracted from pools of 100 pairs of male and female moth antennae using TRIzol RNA extraction reagent (Life Technologies, Carlsbad, CA) according to the manufacturer’s protocol. RNA quality and quantity was determined using an Agilent 2100 bioanalyzer (Agilent Technologies, Santa Clara, CA). High quality total RNA isolated from male and female antennae was used to make libraries for paired-end sequencing on an Illumina HiSeq2000 (Axeq Technologies, Rockville, MD).

Read pair quality control check was carried out using FastQC [43]. Sequence data were then pre-processed by the removal of adapters and trimming to a minimum quality threshold of 20 bp and minimum length of 95 bp using fastq-mcf from the ea-utils package [44]. Thereafter, duplicate reads were removed and trimmed by 15 bases from the 5' end and reads containing Ns or mononucleotides were removed using in-house Perl scripts. *De novo* assembly of the individual libraries was then performed by trans-ABySS (version 1.3.2) [45], where a k-mer series from *k =* 31 to *k* = 75 with an increment of two was used.

### Gene Identification and Phylogenetics

BLAST searchable databases consisting of transcriptomic contigs from male and female *E*. *postvittana* antennae were used to identify candidate chemosensory genes. Tblastn searches were performed with publically available lepidopteran sequences, including previously identified chemosensory genes from *E*. *postvittana* [37–39]. Identified transcripts were imported into Geneious (version 6.0.6) [46] and annotated for open reading frames, start and stop codons and 5' and 3' untranslated regions. The longest contigs identified for a particular gene from male and female antennal transcriptomes were aligned to confirm sequence accuracy.

Predicted amino acid sequences of *E*. *postvittana*, OBP, OR and CXE genes were used in multiple sequence alignments and phylogenetic analyses to confirm their annotation and to examine their relatedness to similar genes from other moths. Datasets for each chemosensory gene family were compiled using publicly available sequences from the silkworm genome database, and GenBank and accession numbers for these can be found in supplementary file, S1 Dataset. Amino acid sequences were aligned using Muscle as implemented within Geneious. Maximum likelihood trees were constructed using MEGA (version 6.0.5) [47], employing the Jones Taylor-Thornton substitution model, and node support was assessed using 1000 bootstrap replicates. Resulting trees were displayed using FigTree (version 1.4.0) [48] and annotated in Adobe Illustrator. Cellular localization of CXEs was predicted using PSORT [49].

### Gene Expression Analyses

Cleaned RNASeq reads were mapped to a known set of olfactory receptor genes using Tophat (version 2.0.13) [50]. The resulting alignments were then used to obtain the Fragments per Kilobase of transcript per Million (FPKM) values using cufflinks (version 2.2.1) [51].

To confirm RNAseq counting results and to compare the expression of candidate ORs in male and female antennae, quantitative RT-PCR (qPCR) was performed using cDNA prepared from three independent biological replicates of pooled male and female antennae and bodies. One of these sets of biological replicates of male and female antennae was the same as that used for the RNAseq experiment described above. RNA extracted from each sample was treated with DNAse I (Life Technologies) and converted to cDNA using the iScript cDNA Synthesis Kit (BioRad, Hercules, CA). cDNA was synthesized from each sample with and without reverse transcriptase to allow for detection of genomic DNA contamination by PCR. cDNA samples that tested negative for genomic DNA contamination were used in qPCR experiments.

Forward and reverse primers for each of the 70 putative *E*. *postvittana* OR genes, as well as the housekeeping genes *actin*, *α-tubulin* and *elongation factor-1α* (*EF-1α*), were designed using Primer 3 implemented within Geneious v6.0.5. Primer pairs were designed to amplify products of 100–200 bp in length, have TMs of 60°C (± 2°C), a G/C content of 40–60% and to have two to three G or C nucleotides on the 3' end.

Quantitative real-time PCR was conducted on a LightCycler 480 II using Syber Green Master Mix (Roche, Basel, Switzerland) under the following reaction conditions: an initial 2-min incubation at 94°C followed by 45 cycles of 94°C for 15 s, then 60°C for 30 s, then 72°C for 30 s. Each primer pair was tested in triplicate against three biological replicates of each cDNA sample, as well as to a no-template control on a single 384-well microtiter plate. The housekeeping genes *actin*, *α-tubulin* and *EF-1α* were tested in triplicate against each biological replicate of each cDNA sample, as well as a no-template control, on each 384-well microtiter plate. The efficiency of each primer pair in each cDNA sample and the Cycle Threshold (Ct) value for each PCR was determined using LinRegPCR (version 11.0) software [52,53]. Amplification of single products was verified by melting curve analysis and electrophoresis. Amplification of target sequences was verified by Sanger sequencing of qPCR products.

The relative expression of each gene was calculated using a modified version of the ∆Ct method [52,54]. Because the efficiency of a given primer set varied between cDNA samples, resulting Ct values for a particular gene and sample were corrected using the formula (E_MAX_) ^Ct^_corrected_ = (E_sample ‘X’_)^Ct^_sample ‘X’_, where E_MAX_ equals the highest efficiency for a primer pair from all samples, E_sample ‘X’_ equals the efficiency of that primer pair in sample ‘X’, Ct_sample ‘X’_ equals the measured Ct value for sample ‘X’, and Ct_corrected_ equals the corrected Ct value for sample ‘X’. A normalization factor was determined for each sample by averaging the corrected Ct values for the three housekeeping genes from that sample. The relative expression of each gene to the normalization factor for each sample was calculated using the formula (E_MAX_)^(∆Ct)^, where E_MAX_ equals the highest efficiency for a particular primer set and ∆Ct equals the difference between the Ct of the primer set in that sample and the normalization factor for that sample. The average relative expression was calculated by averaging the relative expression of each gene in three biological replicates. Significant differences between the relative expressions of a particular gene between male and female antennae were determined using a Welch two-sample T-test.

## Results

### Transcriptome Assembly

Raw read numbers for the female and male transcriptomes were 166,566,716 and 171,208,876, respectively. Individual transcriptome assemblies were generated for female and male antennae. The female antennal assembly generated 270,708 transcripts with an N50 of 1,319 bp and a maximum transcript size of 16,438 bases (GenBank accession SRX456215), whereas the male antennal assembly yielded 266,710 transcripts with an N50 of 1,277 bp and a maximum transcript size of 14,723 bases (GenBank accession SRX456419).

### Identification of Chemosensory Genes and Phylogenetics

In this study a total of 19 IRs, nine GRs, two SNMPs, 34 OBPs, 13 CSPs, 27 CXEs, 20 GSTs, 49 CYPs and 18 TOs were identified from male and female *E*. *postvittana* antennal transcriptomes and their sequences submitted to the Transciptome Shotgun Assembly Sequence Database (TSA) under the accession GCVX00000000. The version described in this paper is the first version, GCVX01000000. The sequences of these genes were confirmed and completed, where possible, using the draft genome of *E*. *postvittana* (unpublished data). DNA and predicted protein sequences for this set of 191 chemosensory genes can be found in supplementary file, S2 dataset; all but six of these sequences (EposCXE36, EposCYP11, EposCYP38, EposCYP48, EposIR4 and EposIR75p) appear to represent the full-length coding sequence of each gene.

Additionally a total of 60 candidate ORs were identified and assembled from the two transcriptome databases, with an extra ten identified from the draft genome bringing the total number of chemosensory genes identified to 261. Sequences for 55 of the 60 ORs identified from the transcriptome have been deposited into TSA under the accession GCVX00000000; this submission does not include two genes, *EposOR9* and *EposOR20* as they contained internal sequence gaps. Also, the three previously identified EposORs from Jordan et al. [38] have been updated in GenBank. The sequences of the 70 *E*. *postvittana* OR genes were confirmed, and the coding region completed where necessary, against the draft genome. Only the open reading frame of two OR genes, *EposOR11* and *EposOR20*, are partial because of missing sequence relating to predicted exon 1 for *EposOR11* and predicted exon 5 for *EposOR20*. DNA and predicted protein sequence data for all 70 ORs can be found in supplementary file, S3 Dataset.

**Fig 1:**
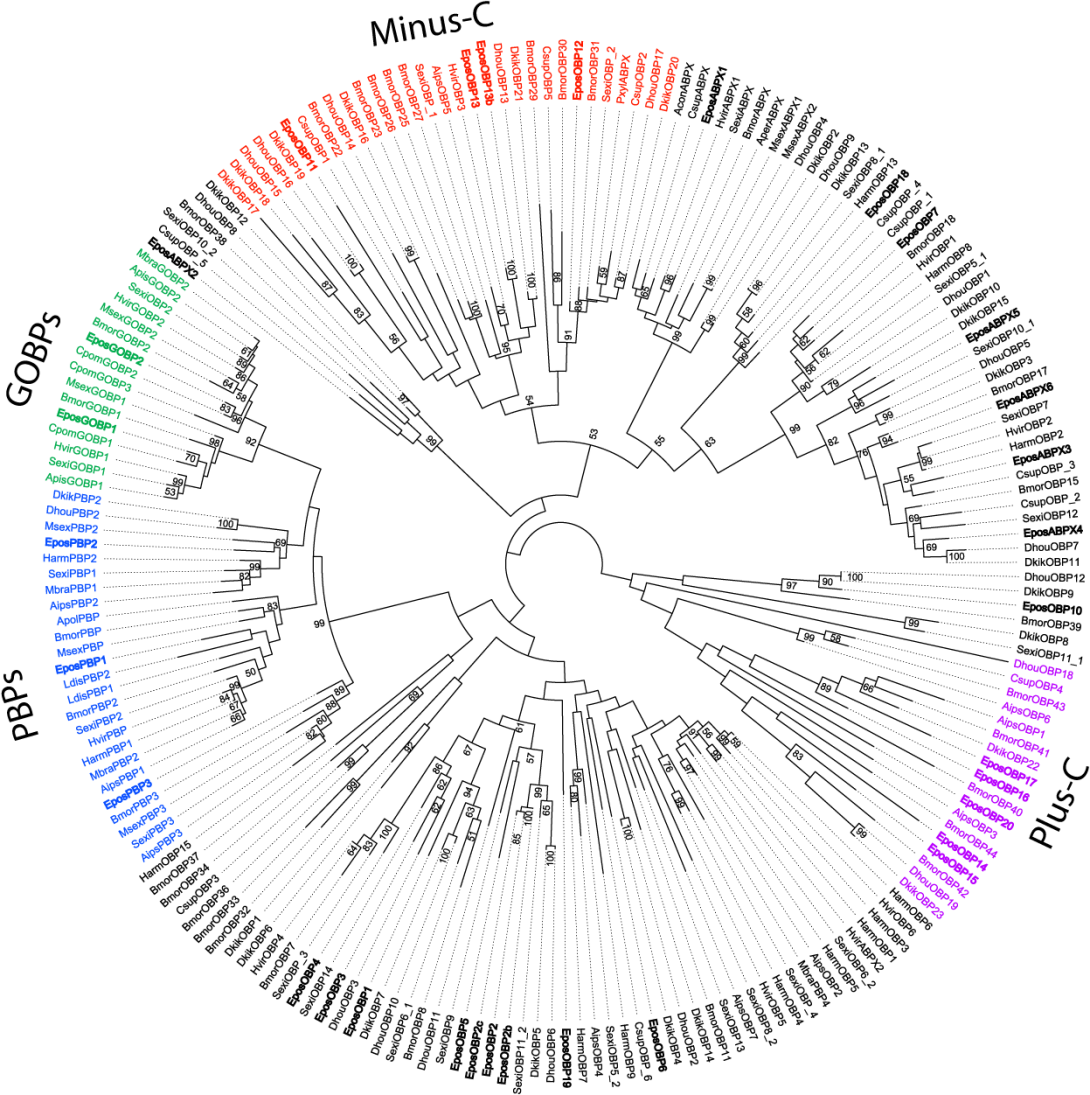
A maximum likelihood phylogenetic tree of odorant binding proteins (OBPs) from moths. Includes sequences from *Epiphyas postvittana* (Epos), *Bombyx mori* (Bmor), *Cydia pomonella* (Cpom), *Chilo suppressalis* (Csup), *Dendrolimus houi* (Dhou), *Dendrolimus kikuchii* (Dkik), *Helicoverpa armigera* (Harm), *Heliothis virescens* (Hvir), *Lymantria dispar* (Ldis), *Mamastra brassicae* (Mbra), *Manduca sexta* (Msex), *Plutella xylostella* (Pxyl) and *Spodoptera exigua* (Sexi). OBPs identified from *E*. *postvittana* are in bold. Node support was assessed using bootstrap replicates of 1000 and values greater than 50% are shown. Sub-groups including the PBPs, GOBPs, minus-Cs and plus-Cs are marked.

**Fig 2:**
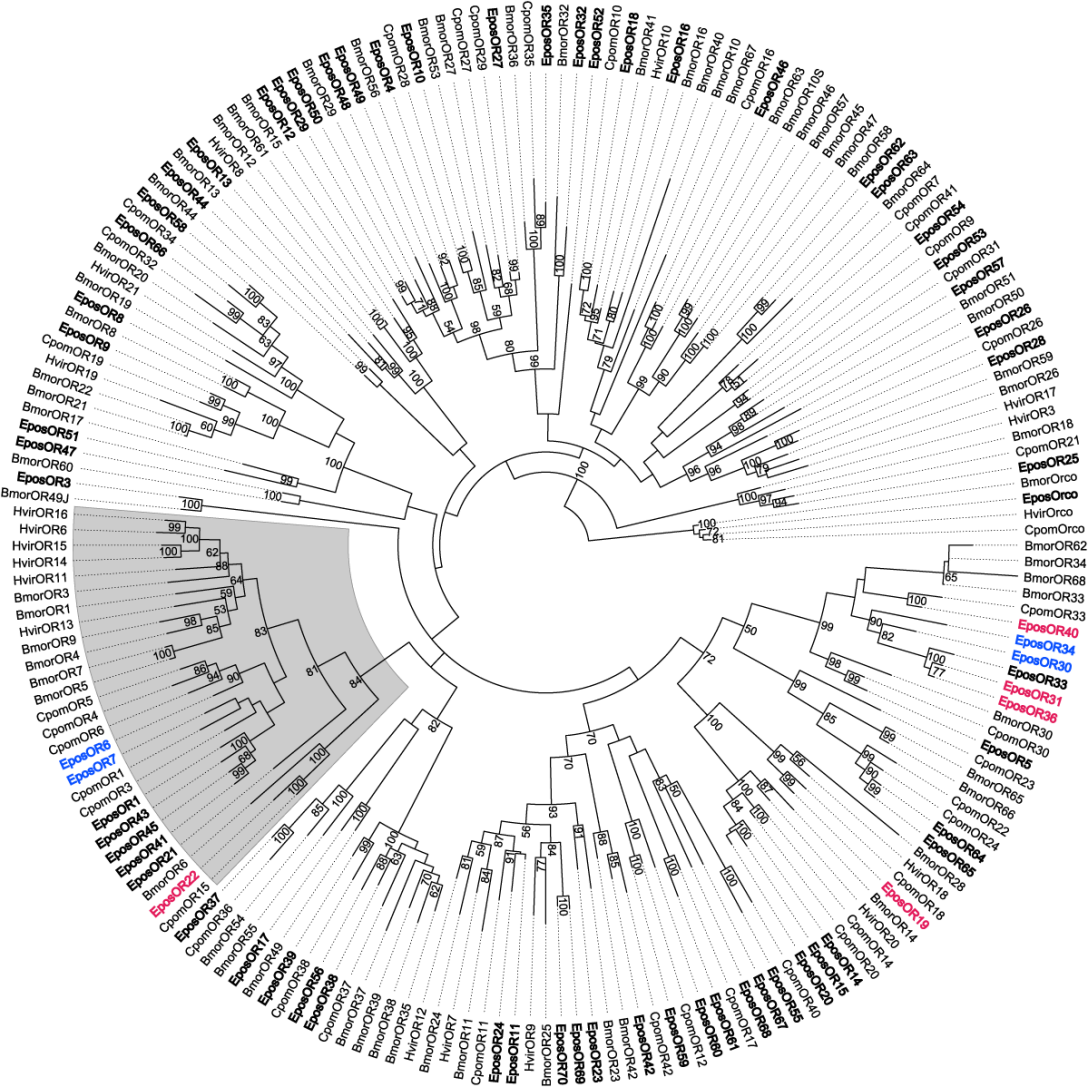
A maximum likelihood phylogenetic tree of odorant receptors (ORs) from moths. Includes sequences from *Epiphyas postvittana* (Epos), *Cydia pomonella* (Cpom), *Heliothis virescens* (Hvir) and *Bombyx mori* (Bmor), and is rooted with the OR co-receptor, Orco. The lepidopteran ‘pheromone receptor’ clade is shaded. *E*. *postvittana* ORs are shown in bold font with male-biased *E*. *postvittana* ORs colored blue and female-biased *E*. *postvittana* ORs colored pink. Node support was assessed using bootstrap replicates of 1000 and values greater than 50% are shown.

**Fig 3:**
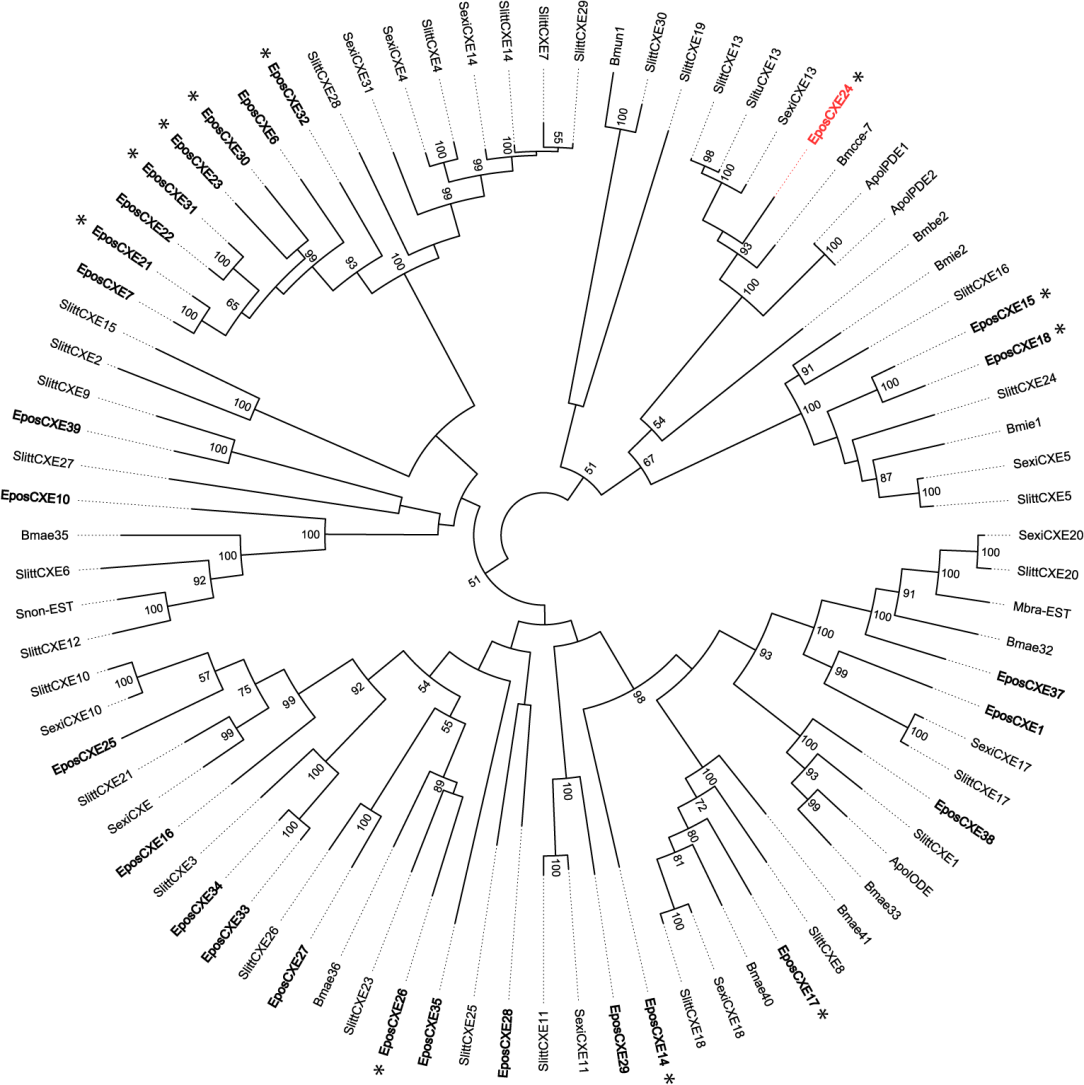
A maximum likelihood phylogenetic tree of antennal carboxylesterases (CXEs) from moths. Includes sequences from *Epiphyas postvittana* (Epos), *Bombyx mori* (Bm), *Antheraea polyphemus* (Apol), *Mamestra brassicae* (Mbra), *Spodoptera littoralis* (Slitt), *Sesamia nonagrioides* (Snon), *Spodoptera litura* (Slitu) and *Spodoptera exigua* (Sexi). CXEs identified from *E*. *postvittana* are in bold and those predicted to be secreted are indicated with an asterisk. The predicted pheromone degrading enzyme is shown in red. Node support was assessed using bootstrap replicates of 1000 and values greater than 50% are shown.

Phylogenetic analyses of the chemosensory gene families thought to be associated with pheromone reception and degradation in *E*. *postvittana*, namely the OBP, OR and CXE, multigene families from antennae, are provided in Figs 1, 2 and 3. The OBP gene family can be split into different sub-groups according to sequence motifs; the first group are PBPs, GOBPs and OBPS that contain six well-conserved cysteines, the second group are OBPs that are missing two of the conserved cysteines, generally at positions 2 and 5 (minus-C group); and the third group are OBPs that contain extra cysteines and a conserved proline (plus-C group) [55,56]. The EposOBPs were compared against OBPs from 16 other lepidopteran species to construct a phylogenetic tree (Fig 1). Two sequences, EposOBP8 and 9, did not align well with the other OBPs and were not found to exhibit many of the motifs associated with insect OBPs and were therefore not included in any further analyses. In the resulting phylogenetic tree, the three EposPBPs fall in with the other moth PBPs, the two EposGOBPs group with the other moth GOBPs, four EposOBPs fall into the minus-C group (EposOBP11, 12, 13 and 13b), five into the plus-C group (EposOBP14, 15, 16, 17 and 20), and the remaining EposOBPs and EposABPXs are distributed across the tree. All the EposOBPs form orthologous clusters with one or many OBPs from other species.

Phylogenetic analysis of the EposORs was performed against comprehensive OR datasets from three other lepidopteran species. Of the 70 *E*. *postvittana* ORs, eight receptors (EposOR1, 6, 7, 21, 22, 41, 43, and 45) fall into a well-supported clade that contains PRs from other moth species, including BmorOR1, BmorOR3, HvirOR6, HvirOR13, HvirOR14 and HvirOR16 [16–18,57,58] (Fig 2). The remaining 62 *E*. *postvittana* ORs are dispersed throughout the phylogeny, often forming orthologous sets with other moth ORs. There are only a few instances of radiations of ORs within the *E*. *postvittana* lineage, including one group of five ORs (EposOR30, 31, 33, 34 and 36), one of four ORs (EposOR1, 41, 43 and 45) that falls within the clade containing pheromone receptors from other species, and another of three ORs (EposOR14, 15 and 20) (Fig 2).

Comparison of the EposCXEs with other moth antennal esterases revealed a number of orthologous sets and one distinct radiation of *E*. *postvittana* genes containing eight sequences (Fig 3). One *E*. *postvittana* CXE, namely EposCXE24, falls into a clade containing pheromone-degrading enzymes (PDEs) from other moths [26]. Eleven EposCXEs, including EposCXE24, were predicted to contain signal peptides and be extracellular in their localization by PSORT, providing evidence for their potential secretion into the sensillum lymph.

### Gene Expression Analyses

Of the 191 olfactory-related genes not including ORs, RNAseq count data revealed normalized transcript counts ranging from 0 to approximately 8.6 x 10^6^ in moth antennae (Table 1). Many of these genes display sex-biased antennal expression patterns; however, the statistical significance of the biased expression could not be assessed because the RNAseq data were conducted without replication.

**Table 1:** Transcript counts in antennal tissue from male and female *Epiphyas postvittana* as determined by RNAseq.

	RNASeq Data (FPKM)			RNASeq Data (FPKM)			RNASeq Data (FPKM)	
Gene	female antennae	male antennae	Gene	female antennae	male antennae	Gene	female antennae	male antennae
**EposABPX1**	194673	305620	**EposCYP21**	34	26	**EposIR1**	21	15
**EposABPX2**	27310	27431	**EposCYP22**	113	66	**EposIR3**	101	66
**EposABPX3**	75729	126520	**EposCYP23**	372	111	**EposIR4**	72	24
**EposABPX4**	21668	19058	**EposCYP24**	240	68	**EposIR7d**	14	7
**EposABPX5**	664	327	**EposCYP25**	4	4	**EposIR8a**	766	504
**EposABPX6**	24780	18822	**EposCYP26**	60	19	**EposIR21a**	53	27
**EposCSP1**	30415	26582	**EposCYP27**	44	24	**EposIR25a**	185	123
**EposCSP2**	51286	17848	**EposCYP28**	42	7	**EposIR41a**	60	35
**EposCSP3**	17920	12770	**EposCYP29**	14	11	**EposIR68**	51	31
**EposCSP4**	98800	80082	**EposCYP30**	88	39	**EposIR75b**	31	16
**EposCSP5**	52504	6969	**EposCYP31**	66	39	**EposIR75d**	10	9
**EposCSP6**	87	123	**EposCYP32**	19	9	**EposIR75e**	44	27
**EposCSP7**	88271	41531	**EposCYP33**	21	12	**EposIR75p**	31	11
**EposCSP8**	122	27	**EposCYP34**	8	13	**EposIR75q.1**	20	11
**EposCSP9**	496	90	**EposCYP35**	4	2	**EposIR75q.2**	295	225
**EposCSP10**	102	16	**EposCYP36**	493	322	**EposIR76b**	341	277
**EposCSP11**	105	69	**EposCYP37**	13	13	**EposIR87a**	127	77
**EposCSP12**	79	26	**EposCYP38**	23	140	**EposIR93a**	4	1
**EposCSP13**	64	12	**EposCYP39**	92	38	**EposOBP1**	1790	673
**EposCXE1**	7	6	**EposCYP40**	6	8	**EposOBP2**	23	13
**EposCXE6**	6	3	**EposCYP41**	38	10	**EposOBP2b**	35	14
**EposCXE7**	13	9	**EposCYP42**	81	30	**EposOBP2c**	165	78
**EposCXE14**	17	17	**EposCYP43**	111	72	**EposOBP3**	5652	1098
**EposCXE15**	59	77	**EposCYP44**	177	87	**EposOBP4**	632	337
**EposCXE16**	105	95	**EposCYP45**	61	34	**EposOBP5**	2909	716
**EposCXE17**	207	101	**EposCYP46**	54	142	**EposOBP6**	203263	67477
**EposCXE18**	39	32	**EposCYP47**	47	12	**EposOBP7**	159629	45042
**EposCXE21**	18	7	**EposCYP48**	119	710	**EposOBP8**	51	19
**EposCXE22**	76	47	**EposCYP49**	491	164	**EposOBP9**	274	114
**EposCXE23**	21	8	**EposCYP50**	15	3	**EposOBP10**	147	45
**EposCXE24**	10	5	**EposCYP51**	40	25	**EposOBP11**	365	11
**EposCXE25**	110	83	**EposGOBP1**	18273	10673	**EposOBP12**	95	32
**EposCXE26**	65	80	**EposGOBP2**	241065	175652	**EposOBP13**	1302	15
**EposCXE27**	15	9	**EposGR1**	8	1	**EposOBP13b**	6895	36
**EposCXE28**	68	23	**EposGR2**	7	5	**EposOBP14**	173	700
**EposCXE29**	19	12	**EposGR3**	3	3	**EposOBP15**	2048	1051
**EposCXE30**	22	9	**EposGR4**	18	8	**EposOBP16**	259	197
**EposCXE31**	12	8	**EposGR5**	18	14	**EposOBP17**	10	19
**EposCXE32**	3	1	**EposGR6**	51	25	**EposOBP18**	317386	78324
**EposCXE33**	24	14	**EposGR7**	64	23	**EposOBP19**	8234	6170
**EposCXE34**	14	12	**EposGR8**	59	32	**EposOBP20**	2113	1243
**EposCXE35**	42	21	**EposGR9**	42	4	**EposPBP1**	97046	863482
**EposCXE36**	5	6	**EposGST2**	332	203	**EposPBP2**	48920	10709
**EposCXE37**	98	53	**EposGST4**	6241	6532	**EposPBP3**	14081	50553
**EposCXE38**	65	39	**EposGST5**	518	372	**EposSNMP1**	855	364
**EposCXE39**	22	15	**EposGST6**	130	81	**EposSNMP2**	501	344
**EposCYP1**	132	87	**EposGST7**	1000	1800	**EposTO1**	103597	50124
**EposCYP3**	61	20	**EposGST8**	1267	472	**EposTO2**	950	834
**EposCYP4**	162	114	**EposGST9**	5264	3256	**EposTO3**	3735	2115
**EposCYP5**	113	65	**EposGST10**	346	202	**EposTO4**	3774	3826
**EposCYP6**	1532	956	**EposGST11**	17621	17056	**EposTO5**	59	49
**EposCYP7**	118	42	**EposGST12**	245	134	**EposTO6**	48	13
**EposCYP9**	535	164	**EposGST13**	708	259	**EposTO7**	60	13
**EposCYP10**	360	107	**EposGST14**	337	412	**EposTO8**	322	38
**EposCYP11**	13	15	**EposGST15**	48	48	**EposTO9**	342	228
**EposCYP12**	223	116	**EposGST16**	190	94	**EposTO10**	402	444
**EposCYP13**	4	6	**EposGST17**	755	552	**EposTO11**	33	7
**EposCYP14**	9519	5579	**EposGST18**	149	85	**EposTO12**	200	83
**EposCYP15**	73	39	**EposGST19**	123	77	**EposTO13**	263	33
**EposCYP16**	60	10	**EposGST20**	406	197	**EposTO14**	2668	2416
**EposCYP17**	44	22	**EposGST21**	59	11	**EposTO15**	74	18
**EposCYP18**	6592	3419	**EposGST22**	69	68	**EposTO16**	14	6
**EposCYP19**	82	17	**EposIgluR**	14	6	**EposTO17**	8	3
**EposCYP20**	1315	732				**EposTO18**	11	7

Antennal binding protein X (APBX), chemosensory protein (CSP), carboxylesterase (CXE), cytochrome p450 (CYP), general odorant binding protein (GOBP), gustatory receptor (GR), glutathione-S-transferase (GST), ionotropic receptor (IR), odorant binding protein (OBP), pheromone binding protein (PBP), sensory neuron membrane protein (SNMP), and take out protein (TO). Data represent transcript counts from a single transcriptome made from male or female antennal mRNA.

Of the 70 *E*. *postvittana* OR genes identified in the antennal transcriptomes or genome, 65 had normalized RNAseq counts greater than one (Table 2). Transcripts for the *EposOR8*, *11*, *13*, *69* and *70* genes were not detected in the male or female antennal transcriptomes by RNAseq analysis. Interestingly, *EposOR8* and *EposOR13* were detectable by PCR using the same RNA starting material from which the transcriptomes were made, which is likely the consequence of the greater sensitivity of qPCR over RNAseq, given the depth of sequencing achieved. This sensitivity difference also likely explains why some gene transcripts were detected in both sexes by qPCR but in only one by RNAseq.

**Table 2:** Odorant receptor (OR) mRNA expression in antennal and body tissue of female and male *Epiphyas postvittana* as determined by quantitative RT-PCR and OR transcript counts in antennal tissue as determined by RNAseq.

	qPCR data (fold change to reference genes)				RNASeq Data (FPKM)	
Gene	female antennae	female body	male antennae	male body	female antennae	male antennae
**EposOR1**	0.03263	0.00024	0.07130	BLD	**191**	**104**
**EposOrco**	2.62100	BLD	3.55188	BLD	**2676**	**1397**
**EposOR3**	0.00768	BLD	0.00663	BLD	**35**	**30**
**EposOR4**	0.03408	BLD	0.03337	0.00011	**72**	**53**
**EposOR5**	0.01009	0.00002	0.00138	BLD	**319**	**37**
**EposOR6**	0.00073	BLD	0.04183	0.00600	**0**	**12**
**EposOR7**	0.00315	0.00010	0.01561	0.00138	**0**	**90**
**EposOR8**	0.00053	BLD	0.00006	0.00002	**0**	**0**
**EposOR9**	0.00145	BLD	0.00127	BLD	**18**	**6**
**EposOR10**	0.00791	0.00007	0.00624	0.00115	**37**	**9**
**EposOR11**	BLD	BLD	BLD	BLD	**0**	**0**
**EposOR12**	0.00143	BLD	0.00192	0.00001	**74**	**74**
**EposOR13**	0.00028	0.00004	0.00071	0.00059	**0**	**0**
**EposOR14**	0.01350	0.00003	0.00650	0.00028	**35**	**12**
**EposOR15**	0.03509	0.00502	0.02606	0.02465	**73**	**34**
**EposOR16**	0.00265	BLD	0.00726	0.00023	**3**	**1**
**EposOR17**	0.00560	0.00493	0.01428	0.00325	**2**	**1**
**EposOR18**	0.03153	0.00029	0.02091	BLD	**63**	**28**
**EposOR19**	0.07996	0.00007	0.03561	BLD	**288**	**57**
**EposOR20**	0.01524	0.00139	0.01714	0.00381	**74**	**23**
**EposOR21**	0.00729	0.00002	0.00099	0.00003	**66**	**6**
**EposOR22**	0.07837	0.00007	0.02709	BLD	**108**	**43**
**EposOR23**	0.00058	0.00025	0.00145	0.00108	**1**	**1**
**EposOR24**	0.01978	BLD	0.02527	BLD	**20**	**9**
**EposOR25**	0.03330	0.00034	0.03440	0.00260	**46**	**15**
**EposOR26**	0.05425	0.01337	0.11274	0.01719	**21**	**15**
**EposOR27**	0.05022	BLD	0.04528	0.00128	**104**	**46**
**EposOR28**	0.01670	BLD	0.00903	0.00009	**22**	**20**
**EposOR29**	0.04849	0.00020	0.02772	0.00027	**36**	**18**
**EposOR30**	0.02020	0.00398	0.69629	0.02740	**0**	**536**
**EposOR31**	0.00616	BLD	0.00003	BLD	**189**	**0**
**EposOR32**	0.04552	BLD	0.03881	BLD	**18**	**6**
**EposOR33**	0.00640	BLD	0.00047	0.00004	**60**	**0**
**EposOR34**	0.01131	0.00043	0.32708	0.00233	**1**	**779**
**EposOR35**	0.00408	BLD	0.00543	BLD	**26**	**14**
**EposOR36**	0.02528	BLD	0.00014	0.00004	**57**	**0**
**EposOR37**	0.00393	0.00008	0.01092	0.00008	**20**	**17**
**EposOR38**	0.04631	0.00006	0.11024	0.00197	**50**	**19**
**EposOR39**	0.09887	BLD	0.19689	BLD	**50**	**24**
**EposOR40**	0.32466	0.00007	0.00507	0.00135	**383**	**1**
**EposOR41**	0.00446	0.00019	0.00911	BLD	**29**	**19**
**EposOR42**	0.05713	BLD	0.09239	BLD	**70**	**47**
**EposOR43**	0.09436	0.00068	0.04906	0.00181	**21**	**17**
**EposOR44**	0.00199	BLD	0.00317	BLD	**44**	**19**
**EposOR45**	0.01907	0.00015	0.02347	0.00011	**147**	**75**
**EposOR46**	0.00932	0.00015	0.01168	0.00079	**28**	**16**
**EposOR47**	0.08845	0.00096	0.07746	0.00430	**52**	**31**
**EposOR48**	0.06465	0.00007	0.07733	0.00034	**102**	**75**
**EposOR49**	0.00271	0.00022	0.00522	0.00008	**6**	**5**
**EposOR50**	0.00054	0.00008	0.00144	0.00010	**3**	**1**
**EposOR51**	0.05788	0.00038	0.06611	0.00031	**163**	**84**
**EposOR52**	0.00986	BLD	0.00955	BLD	**24**	**9**
**EposOR53**	0.01144	0.00003	0.01077	0.00011	**128**	**92**
**EposOR54**	0.02487	0.00003	0.02158	0.00011	**47**	**23**
**EposOR55**	0.00098	0.00001	0.00569	0.00026	**37**	**28**
**EposOR56**	0.00100	BLD	0.00071	BLD	**10**	**4**
**EposOR57**	0.00114	BLD	0.00610	0.00001	**8**	**3**
**EposOR58**	0.00405	0.00001	0.00339	0.00003	**81**	**40**
**EposOR59**	0.01240	0.00061	0.00962	0.00044	**11**	**3**
**EposOR60**	0.00464	0.00010	0.01035	BLD	**38**	**32**
**EposOR61**	0.01409	0.00006	0.01719	BLD	**160**	**50**
**EposOR62**	0.00589	BLD	0.00451	BLD	**30**	**16**
**EposOR63**	0.00017	BLD	0.00011	BLD	**7**	**3**
**EposOR64**	0.05393	BLD	0.01088	BLD	**227**	**56**
**EposOR65**	0.01115	0.00005	0.00184	0.00004	**108**	**17**
**EposOR66**	0.00506	0.00020	0.00296	BLD	**14**	**4**
**EposOR67**	0.02124	0.00001	0.01068	0.00007	**128**	**43**
**EposOR68**	0.02752	0.00027	0.02945	BLD	**55**	**32**
**EposOR69**	BLD	BLD	BLD	BLD	**0**	**0**
**EposOR70**	BLD	BLD	BLD	BLD	**1**	**0**

Quantitative RT-PCR data represent the mean fold change to reference genes from three biological replicates. RNAseq data represent transcript counts from a single transcriptome made from male or female antennal mRNA. BLD = Below Limit of Detection.

**Fig 4:**
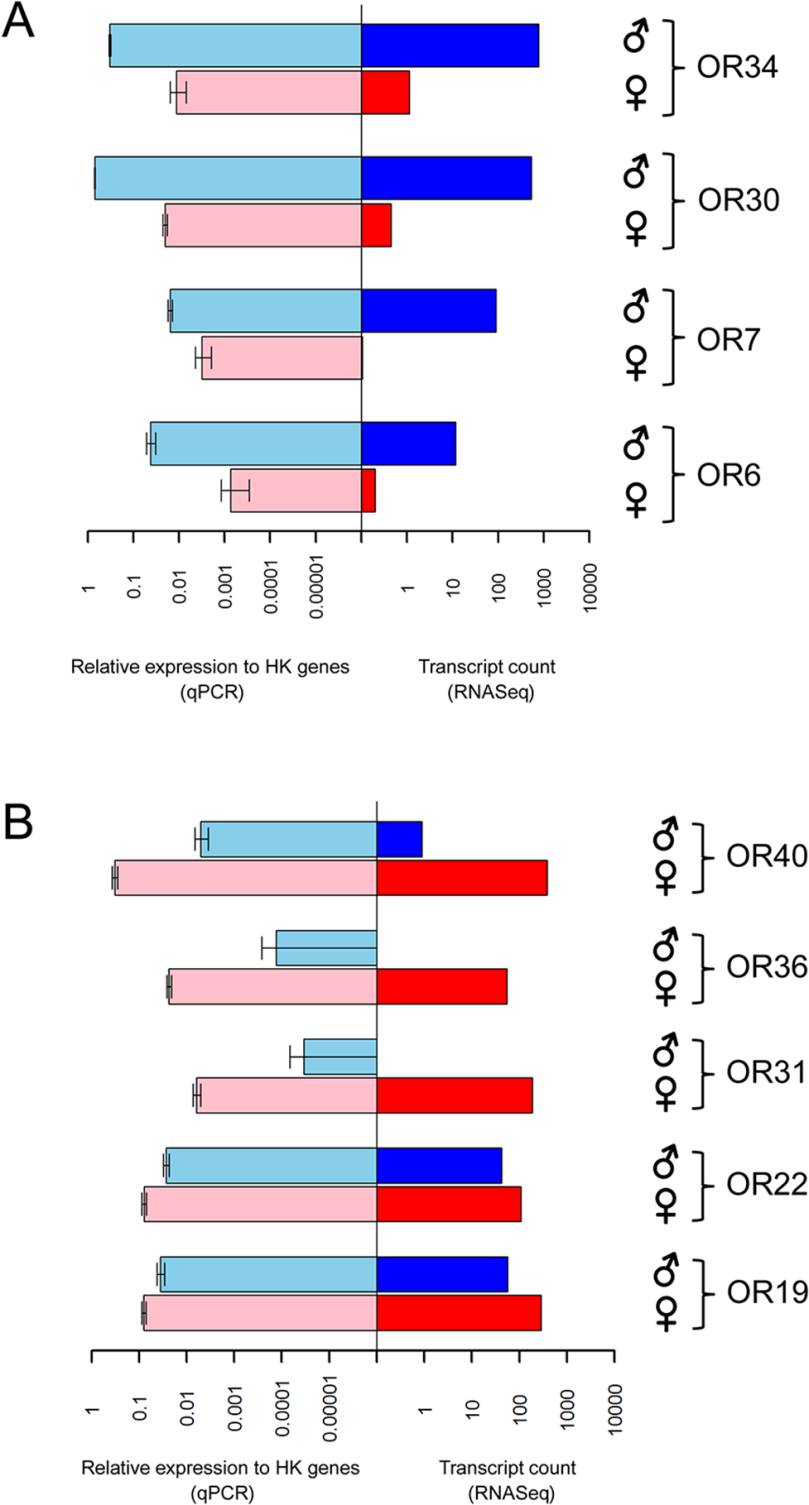
Male-biased (A) and female-biased (B) antennal odorant receptor mRNA expression in *Epiphyas postvittana*. Comparison of OR mRNA expression in male (♂) and female (♀) antennae as determined by quantitative real-time PCR (qPCR; left panel) and RNASeq (right panel). For qPCR data, bars represent mean (± SEM) of OR mRNA expression relative to reference genes from three biological replicates. For RNAseq data, bars represent transcript counts from a single transcriptome made from male or female antennal mRNA.

Sixty-seven of the 70 *E*. *postvittana* OR genes were detected by qPCR for expression in adult male or female antennae or bodies. Transcripts for *EposOR11*, *69* and *70* were not detected in any tissues despite attempts with multiple primer pairs. Of the 67 OR transcripts that were detected, 64 showed enriched expression in antennae compared to bodies, with 30 detected exclusively in male and/or female antennae and not in male or female bodies. Four genes, *EposOR6*, *7*, *30* and *34* had significantly greater levels of mRNA expression in male than in female antennae (*P* = 0.046, *P* = 0.009, *P* = 0.004 and *P* = 0.002, respectively) (Fig 4A). Five receptors, *EposOR19*, *22*, *31*, *36* and *40* had significantly greater levels of mRNA expression in female than in male antennae (*P* = 0.023, *P* = 0.032, *P* = 0.031, *P* = 0.012 and *P* = 0.018, respectively) (Fig 4B). We note that the EposOR genes that showed significant sex-biased mRNA expression by qPCR also showed biased expression patterns by RNAseq, demonstrating that despite the fact that we could not conduct statistical analyses on RNAseq data, it was a good predictor of sex-biased expression for these genes.

## Discussion

Transcriptome sequencing has become a popular approach to identify genes involved in tissue-specific functions. Here we have used this approach to identify genes involved in the peripheral events of odorant and pheromone reception in the lepidopteran pest, *E*. *postvittana*. In total, 266,710 and 270,708 putative transcripts were produced from male and female antennae, respectively. From these we inferred the presence of 251 predicted proteins that are potentially involved in odorant and pheromone binding, reception and degradation. The average transcript length of our male (N50 = 1,277 bp) and female (N50 = 1,319 bp) antennal transcriptomes aided the determination of the full length coding sequences of most of these genes through examination of untranslated regions and open reading frames of single contigs. To date we have verified the full length sequence of 36 genes from the dataset, including 30 ORs, three OBPs, two SNMPs and one CXE by PCR from antennal cDNA.

The set of olfactory-related genes that have been identified from the antennae of *E*. *postvittana* has been substantially extended by this study. Previously through micro-sequencing of purified protein and degenerate PCR, four OBPs, EposPBP1, EposPBP2, EposGOBP1 and EposGOBP2, were identified [40]. EST libraries from male antennae were also generated, enabling the identification of sequences for 12 more candidate binding proteins, one takeout protein, three ORs, two SNMPs and 25 odor-degrading enzymes [37]. With this study we have expanded the total number of identified olfactory-related genes from *E*. *postvittana* that are in the public domain to: 34 OBPs, 13 CSPs, 70 ORs, 19 IRs, two SNMPs, 38 CXEs, 22 GSTs, 51 CYPs and 18 TOs. Within the Tortricidae there are few transcriptome datasets from olfactory tissues, with the only other comparable dataset from the antennae of codling moth, *Cydia pomonella* [59]. This *E*. *postvittana* antennal dataset therefore makes an important contribution to the growing datasets of gene sequences from pest tortricids and, in particular genes encoding proteins predicted to be involved in peripheral olfactory signal transduction.

We then focused on genes likely to encode elements of the sex pheromone signal transduction pathway, namely members of the specialized odorant-binding protein family, odorant receptor family, sensory neuron membrane protein family, and carboxylesterase family, since the predominant sex pheromone components of *E*. *postvittana* are acetate esters.

Three pheromone-binding proteins have previously been identified in *E*. *postvittana* [38], with one shown to bind the major sex pheromone component [40]. In this current study no further potential pheromone binding proteins were identified.

Seventy ORs were identified from *E*. *postvittana* antennae in this study, which correlates well with the number of glomeruli (50–70) observed in the antennal lobe of other tortricid moths [60,61]. Odorant receptors that may play a role in pheromone detection are typically identified through either being members of the so-called pheromone receptor clade or by showing male-biased expression in adult antennae. Of the 70 *E*. *postvittana* ORs, eight were identified as being part of the phylogenetic clade associated with a role in pheromone reception in other moths. Seven of the eight EposORs that fall within this ‘PR clade’ contain the characteristic ‘PWE’ amino acid motif within the final transmembrane domain that is seen in members of this group. One receptor, EposOR21, does not contain this motif and phylogenetic analysis suggests this receptor is basal to other receptors in the clade. EposORs 1, 41, 43 and 45 share relatively high (~61%) identity at the protein level and form their own group within the clade, and may have arisen by recent gene duplication events.

The two male-biased EposORs within the PR clade, EposOR6 and EposOR7, are most closely related to ORs from *C*. *pomonella*, CpomOR6 and CpomOR1, respectively. The other receptor that falls in the pheromone receptor clade, EposOR22, is female-biased and is closely related to CpomOR15 [59]. In terms of male-biased expression, EposOR6 and EposOR7 from within the PR clade and EposOR30 and EposOR34 from a more distant part of the phylogenetic tree all showed significantly greater levels of expression in male than in female antennae. This is the first case we are aware of where receptors from outside the PR clade display male-biased expression. These two groups of male-biased receptors (EposOR6 and EposOR7; EposOR30 and EposOR34) form clusters of genes physically within the genome (unpublished draft genome and BAC analysis).

One possible explanation for the observed male-biased expression profiles of EposOR6,7,30 and 34 is that male *E*. *postvittana* have large numbers of tricoid sensilla present on their antennae that are not found on the female [37]. Single sensillum recordings have shown that these tricoid sensilla are responsive to components of the female-produced sex pheromone and therefore are likely to contain olfactory sensory neurons that express PRs [35]. Aside from simple numerical differences in the number of pheromone sensitive sensilla, it is also possible that there are absolute differences in steady state transcript levels of PRs between the sexes in certain classes of olfactory sensory neurons tuned for pheromone. It is difficult to ascertain the relative contribution of these different hypotheses from the methods we employed to assess sex-biased expression from RNA extractions of whole antennae.

In addition to male-biased receptors, we also identified receptors that showed female-biased expression that could potentially be involved in host location or male pheromone recognition. These five female-biased receptors (EposOR19, 22, 31, 36, 40) include two (EposOR31 and EposOR36) that are closely related to the two male-biased ORs that are not part of the PR clade. None of the female-biased receptors was closely related to those found to be female-biased in their expression in *Bombyx mori* (BmorOR19, 30, 45, 46, 47) [62,63]. Functional characterization of these male- and female-biased ORs, as well as members of the PR clade, remains to be conducted.

Two SNMPs were identified among the antennal transcripts of *E*. *postvittana*. In other insects SNMP1 has been implicated in pheromone reception, through its requirement for pheromone reception in *Drosophila* [64] and its ability to enhance the response of ectopically expressed moth pheromone receptors *in vivo* [65]. SNMPs are members of the CD36-like family and may have roles in presentation of the pheromone to the receptor or signal resetting through transporting the ligand inside the cell after receptor activation [66,67].

Twenty-four CXEs were found among the antennal transcripts, with thirteen of them predicted to be secreted. Only one CXE seems a good candidate for being involved in the degradation of *E*. *postvittana* acetate pheromone components based on phylogenetic relatedness to other pheromone-degrading enzymes [26,68], namely EposCXE24. Other PDEs display high affinity for pheromone components and rapid hydrolytic properties [26,69,70] and we would expect similar characteristics for a PDE in *E*. *postvittana* to allow rapid resetting of the pheromone transduction system.

Together this set of genes potentially involved in pheromone and odorant signal transduction in *E*. *postvittana* comprises an important dataset from which to study chemoreception in this organism, as well as a potential set of targets against which to develop novel olfactory-based control strategies for horticultural pests.

## Supporting Information

S1 Dataset Datasets used in the phylogenetic analysis for OBP, OR and CXE gene families. (XLSX)

S2 Dataset DNA and predicted protein sequence for *Epiphyas postvittana* non-OR chemosensory genes. (TXT)

S3 Dataset DNA and predicted protein sequence for *Epiphyas postvittana* OR genes. (TXT)
